# αCP binding to a cytosine-rich subset of polypyrimidine tracts drives a novel pathway of cassette exon splicing in the mammalian transcriptome

**DOI:** 10.1093/nar/gkw088

**Published:** 2016-02-20

**Authors:** Xinjun Ji, Juw Won Park, Emad Bahrami-Samani, Lan Lin, Christopher Duncan-Lewis, Gordon Pherribo, Yi Xing, Stephen A. Liebhaber

**Affiliations:** 1 Department of Genetics, Perelman School of Medicine, University of Pennsylvania, Philadelphia, PA 19104, USA; 2 Department of Microbiology, Immunology, and Molecular Genetics, University of California, Los Angeles, Los Angeles, CA 90095, USA; 3 Department of Computer Engineering and Computer Science, University of Louisville, Louisville, KY 40292, USA; 4 KBRIN Bioinformatics Core, University of Louisville, Louisville, KY 40202, USA; 5 Department of Medicine, Perelman School of Medicine, University of Pennsylvania, Philadelphia, PA 19104, USA

## Abstract

Alternative splicing (AS) is a robust generator of mammalian transcriptome complexity. Splice site specification is controlled by interactions of *cis*-acting determinants on a transcript with specific RNA binding proteins. These interactions are frequently localized to the intronic U-rich polypyrimidine tracts (PPT) located 5′ to the majority of splice acceptor junctions. αCPs (also referred to as polyC-binding proteins (PCBPs) and hnRNPEs) comprise a subset of KH-domain proteins with high affinity and specificity for C-rich polypyrimidine motifs. Here, we demonstrate that αCPs promote the splicing of a defined subset of cassette exons *via* binding to a C-rich subset of polypyrimidine tracts located 5′ to the αCP-enhanced exonic segments. This enhancement of splice acceptor activity is linked to interactions of αCPs with the U2 snRNP complex and may be mediated by cooperative interactions with the canonical polypyrimidine tract binding protein, U2AF65. Analysis of αCP-targeted exons predicts a substantial impact on fundamental cell functions. These findings lead us to conclude that the αCPs play a direct and global role in modulating the splicing activity and inclusion of an array of cassette exons, thus driving a novel pathway of splice site regulation within the mammalian transcriptome.

## INTRODUCTION

RNA splicing is a highly dynamic process that involves as many as 200 protein factors interacting with target sites on a PolII transcript. While many of these RNA-protein (RNP) interactions have been described in detail, the broad array of alternative splicing (AS) events detected in mammalian cells (1,2) and the large number of RNA binding proteins (RBPs) encoded by the mammalian genome, suggest that numerous additional determinants of splicing controls remain to be identified and characterized (3–7). Many of the relevant RBPs (at least 141) compose core components of the mammalian spliceosome complexes (3,8–10) that interact with splice donor and splice acceptor regions and catalyze intron excision and exon ligation (8). The ‘strength’ of a splice acceptor site is impacted by the assembly of RNP complexes at a polypyrimidine tract (PPT) located immediately 5′ of the AG splicing acceptor site. This PPT characteristically consists of a loosely defined U-rich sequence with interspersed C residues (8,11–14). The splicing factor U2AF65 binds directly to this U-rich PPT and recruits its heterodimeric partner U2AF35 and the U2 snRNP complex to the splicing branch site with the initiation of the first of two trans-esterification reactions (8).

Control of splice acceptor activity can be mediated by altering the efficiency and/or productivity of the interactions between U2AF65 and the PPT (8). For example, the RNA binding proteins PTB and hnRNPC have been proposed to repress splice acceptor utilization by blocking the binding and/or activity of U2AF65 at the PPT (11,14,15). The mechanistic details of repression by these two factors appear to be distinct and remain to be fully defined (11,14,15). Recent studies indicate that RBFOX2 protein regulates binding of the early intron recognition factors U2AF and the U1 small nuclear ribonucleoprotein complex (snRNP) (16). Additional sets of RNA binding proteins further contribute to activating or repressing controls over AS. Many of these proteins may be widely expressed while others may mediate regulation of splicing programs in specific cell types (e.g. ESRP family and Nova family (17–21)).

The αCP proteins (also referred to as polyC-binding proteins (PCBPs) and hnRNPEs) comprise a family of RBPs encoded at four dispersed loci (22). These widely expressed proteins contain three highly conserved RNA binding KH domains and shuttle between the nucleus and the cytoplasm (23). The two most highly expressed of these proteins, αCP1 and αCP2, are present in all metazoan organisms studied to date. αCPs have been demonstrated to regulate mRNA stability (24–26) and translation (27,28) in the cytoplasm and exert a global impact on 3′ processing of transcripts in the nucleus (29,30). Several studies have reported an additional impact of αCPs on splicing of specific transcripts encoding CD44 (31–33), Tau (34), CD45 (35) and human (h) α-globin (36,37).

In the current study, we define a global impact of αCPs on alternative splicing. The data reveal that the αCPs selectively enhance the splicing (inclusion) of a defined subset of cassette exons in the human transcriptome. These cassette exons targeted by αCPs are characterized by a cytosine-rich subset of PPTs adjacent to their splice acceptor sites. The demonstrated interactions of αCPs with U2 spliceosome-associated proteins and with U2AF65 are likely to support their observed splicing functions. These findings lead us to conclude that αCP interactions with C-rich PPTs play a significant role in the AS of a subset of cassette exons.

## MATERIALS AND METHODS

### Cell culture and siRNA/shRNA transfection

**Figure 1.**
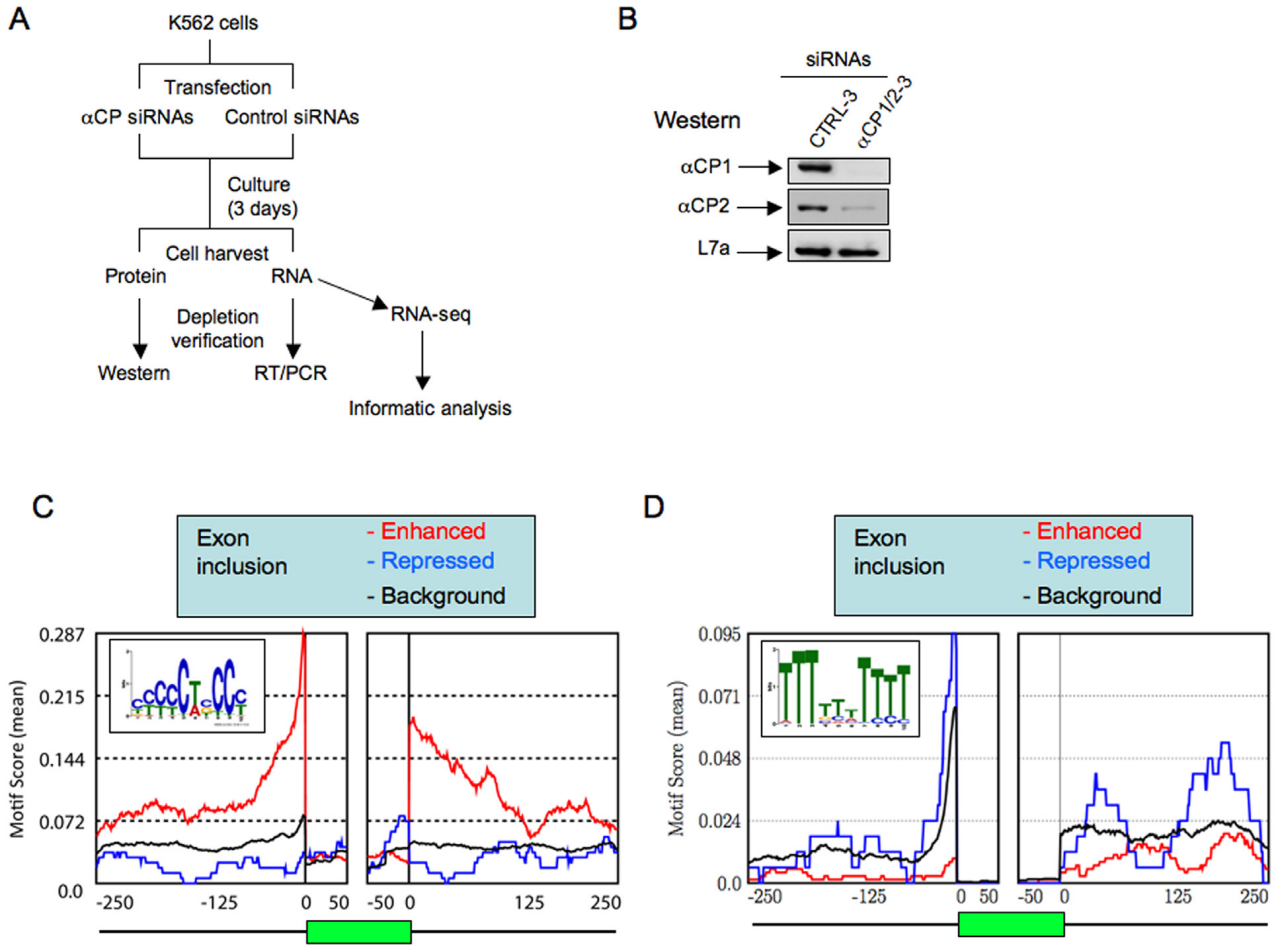
Co-depletion of αCP1/2 in K562 cells enhances splicing of a subset of cassette exons that are enriched for C-rich splice junctions. (**A**) Assessing the impact of αCP1/2 co-depletion on cassette exon splicing; work-flow schematic. Three pools of K562 cells were co-depleted of αCP1 and αCP2 *via* transfection with siRNAs that co-target both encoding mRNAs (αCP1/2 siRNA). Three cell pools transfected with control siRNAs served as parallel controls (see Materials and Methods). Three days post-transfection αCP1/2 depletion was confirmed at the levels of mRNA and protein and the transcriptomes from the depleted and control cell pools were analyzed by RNA-seq. RNA-seq data in these control and αCP1/2 co-depleted transcriptomes were compared by informatic approaches to detect alterations in transcript splicing (see text). (**B**) Co-depletion of αCP1 and αCP2 proteins from K562 cells. K562 cells were transfected with siRNA αCP(1/2)-3 co-targeting αCP1 and αCP2 or with scrambled sequence control siRNA (CTRL-3). Three days post transfection the cells were harvested and assayed by Western blot with antibodies specific to αCP1 and αCP2. Gel loading was assessed using an antibody to ribosomal protein L7a (45). (**C**) Motif analysis of cassette exons whose inclusion was impacted in αCP1/2 co-depleted cells. Cassette exons impacted in cells co-depleted of αCP1/2 were identified by comparative RNA-seq (Table 1). The regions 250 bases ‘upstream’ or ‘downstream’ of each impacted cassette exon in each of the two categories was assessed by MEME (v4.9.0) for conserved motifs. The level of significance was determined by comparison to a ‘Background’ set of 4527 non-impacted alternative exons (rMATS FDR > 50%) in highly expressed genes (FPKM > 5.0). A C-rich motif (shown in the inset) was specifically identified flanking the αCP1/2-enhanced exons (i.e. those repressed by αCP1/2 co-depletion) in contrast to exons repressed or unaffected (Background). The graph shows the positional distribution of this C-rich motif across the regions 5′ and 3′ to the cassette exon (green rectangle). The analysis was executed on 50 bp bins across the indicated regions in each of the three exon groups (αCP1/2-enhanced, αCP1/2-repressed, background). (**D**) Motif analysis of exons whose inclusion was not impacted in αCP1/2 co-depleted cells. Analysis as in (C). The graph shows the positional distribution of the identified U-rich MEME consensus motif (shown in the insert) as assessed by analysis of 50 bp bins across the indicated regions in each of the three exon groups (αCP1/2-enhanced, αCP1/2-repressed, background).

K562 cells were cultured in RPMI 1640 medium, supplemented with 10% fetal bovine serum (FBS) (HyClone) and antibiotic/antimycotic at 37°C in a 5% CO_2_ incubator. K562 cells were transfected with αCP siRNAs or shRNAs using Nucleofector V (Amaxa) as previously described (30). The two αCP1/2 co-depletion siRNAs are previously reported (30). siRNAs to U2AF65 (HSS117616, HSS117617, HSS117618); siRNAs to αCP1 (HSS107632, HSS107633, HSS107634); siRNAs to αCP2 siRNAs (HSS143239, HSS143241, HSS181787) and negative control siRNA (medium GC content) were purchased from Invitrogen. The αCP(1/2)-2 siRNA (30), which targets the 3′ UTR of native αCP1/2 mRNA, was converted to shRNA based on pGFP-V-RS parental vector (Origene Technologies, Inc.). These shRNAs was co-transfected along with scrambled control shRNAs in a subset of studies (Figures 3C and D, and 4B). The siRNA sequences for Figure 1B are shown in Table 3.

**Table 1.** αCP1/2 co-depletion impacts on splicing of a subset of exons

**(A)** Alternative splicing events: αCP KD. versus CTRL (rMATS 3.0.7, hg19, Ensembl release 65 GTF)			
AS events		Significant events FDR <5%, |ΔPSI| ≥ 5%	
SE (Skipped exon)		1051	
MXE (mutually exclusive exon)		168	
A5SS (Alternative 5′ splice site)		56	
A3SS (Alternative 3′ splice site)		85	
RI (Retained intron)		191	
**Total**		**1551**	
(**B**) Distributions of C-rich motifs flanking αCP1/2 enhanced cassette exons (749)			
C-rich motif		3′ of exon	
		(+)	(−)
5′ of exon	(+)	311	333
	(−)	42	63

**(A**) Five categories of AS impacted by αCP1/2 co-depletion are determined by MATS analysis of RNA-seq data sets. The numbers of AS events impacted either positively or negatively by αCP1/2 depletion are summarized for each of the 5 categories. These data were accrued by rMATS analysis at the indicated ΔPSI as described (**Materials** and **Methods)**. (**B**) Distributions of C-rich motifs flanking 749 cassette exons (Skipped exon) whose inclusion was depressed in αCP1/2 co-depleted cells.

**Table 2.** Ingenuity pathway analysis of genes with significant changes (positive or negative) in exon inclusion subsequent to αCP depletion

**(A)** Ingenuity pathway analysis of genes with significant exon inclusion level change (enhanced or inhibited) subsequent to αCP depletion		
Molecular and cellular functions	*P*-value	Number of genes
Cell death and survival	9.62E-03 - 2.08E-11	292
Gene expression	7.71E-04 - 6.46E-11	201
Cellular growth and proliferation	1.00E-02 - 1.41E-10	290
Cell cycle	9.77E-03 - 3.74E-07	139
RNA post-transcriptional modification	2.71E-03 - 1.13E-05	35
(**B**) Ingenuity pathway analysis of genes from 644 cassette exons with 5′ PPT C-rich motif whose inclusion is enhanced by αCPs		
Molecular and cellular functions	*P*-value	Number of genes
Cell death and survival	2.19E-02 - 3.45E-07	157
Cell cycle	2.19E-02 - 1.04E-05	53
Gene expression	2.19E-02 - 2.21E-05	100
Cellular assembly and organization	2.19E-02 - 2.99E-05	111
Cell morphology	2.19E-02 - 3.47E-05	104

**(A)** Ingenuity pathway analysis of genes with significant exon inclusion level change (enhanced or inhibited) subsequent to αCP depletion. (**B**) Ingenuity pathway analysis of genes from 644 cassette exons with 5′ PPT C-rich motif. A total of 333 of the cassette exons whose inclusion is enhanced by αCP1/2 contain the C-rich solely upstream position and 311 contain the C-rich motif both upstream and downstream of the target exon.

**Table 3.** Primers used in the RT-PCR validation studies. Refer to Material and Methods for siRNAs and primers not included in this table

Gene name for RT-PCR	Forward/Reverse primer sequence
VKORC1	CTTTGCCTGACGGGCTTAGT / CCAGGTAGACAGAACCAGCG
WNK4	GCTGCGTAAAGCAAGGGAAT / ACGTGGAATGGATAGGGCAA
ARHGAP4	TGGATCCTCCAGGGGACAAA / AATCAAGCACATCCCCGTCA
Stat2	TGCTCTCAGTTGGCAGTTCT / ATGGTAACTTGCCAGGAGGG
AP1G2	AGCTGGCCAAACTGCTCTAC / GTGCTCAAAGTGCACAAGGC
Human CDK2	GCTTTTGGAGTCCCTGTTCG / GGTCCCCAGAGTCCGAAAGA
TARS2	CCAACTGCCCCTGAGATTTG / TCTTTGGCGTATTCCTCTTGCT
SH2B1	CACCCCATCCCTTTGGAGTC / GTCCATGAAGGGGGTTCAGG
TRPT1	GCATGCGGTCCCATTGTGAA / GCTTGGGGCTACTCTGACAC
TFR2	AGCGGCTTTGGGGTCTATTC / TAGAGTCTGCCCTGGTGGAA
CTTN	TGGTGTGCAGACAGACAGAC / CCATACTTCCCGCCGAATCC
siRNA for αCPs (Figure 1B)	sequence
αCP1/2-3	GUG AAA GGC UAU UGG GCA A
CTRL-3	siGENOME Non-Targeting siRNA #2, Dharmacon, Inc

### RNA-seq analysis

RNA-seq libraries were constructed using the TruSeq RNA Sample Preparation Kit V1 according to manufacturer's instruction. The libraries corresponded to the three control siRNA depletions and three αCP1/2 co-depletions. These six samples comprised four samples used and validated in our previous analysis of 3′ processing (Figure 1, Ji, 2013 MCB (30)) and additional two siRNA treated K562 samples, one control siRNA (CTRL-3: non-targetting, Dharmacon, Inc) and one αCP1/2 siRNA(αCP1/2–3, same as (αCP1/2–1 in (30)) (Figure 1B). These libraries were multiplexed and sequenced on a single channel on the Hiseq-2000 platform using a 100 nt paired-end sequencing protocol by NGSC at University of Pennsylvania (https://ngsc.med.upenn.edu/).

### Alternative splicing analysis

RNA-seq reads were mapped to the human transcriptome (Ensembl, release 65) and genome (hg19) using the software Tophat (38) (v1.4.1) allowing up to 3 bp mismatches per read and up to 2 bp mismatches per 25 bp seed. αCP-regulated differential AS events corresponding to five major types of AS patterns were identified by rMATS (v3.0.7) (39,40) (http://rnaseq-mats.sourceforge.net/) (Table 1). For each AS event, both the reads mapped to the exon–exon junction and the reads mapped to the exon body were used as rMATS input. Putative αCP-regulated AS events were identified as those with significant difference in inclusion levels (|ΔPSI|≥5%) between knockdown and control at an FDR < 5%.

### Motif enrichment analysis

Motifs that were significantly enriched in differential exon skipping events between the αCP depleted and control samples were identified by comparison to background (non-regulated) alternative exons. A total of 4527 alternative exons without splicing changes (rMATS FDR > 50%) in highly expressed genes (FPKM > 5.0 in at least one sample group) were treated as a background exon set. RNA-seq based gene expression levels (FPKM) were calculated by Cuffdiff (v2.2.0) (41). A MEME analysis (v4.9.0) was carried out to detect enriched motifs in a 500 bp window centered on the splice acceptor and splice donor sites (42). To examine the enrichment of identified motifs in the vicinity of the αCP regulated exons, the 500 bp windows were analyzed as 50 bp bins with step-size of 1 bp and the occurrence of each motif within each bin was assessed for positional distribution of each motif.

### Ingenuity pathway analysis

The enrichment analyses of biological functions were generated through the standard use of the IPA package (Ingenuity® Systems). Using the Ingenuity Knowledge Base as a reference set, this analysis identified the most significant biological functions associated with the set of genes that showed significant changes in exon inclusion following αCP depletion. Right-tailed Fisher's exact test was used to calculate the *P*-values determining the probability of each function assigned to the tested data set is due to chance alone.

### Accession number

All RNA-seq data were deposited to the NCBI Gene Expression Omnibus (43) under the accession number GSE71569. The U2AF65 eCLIP data set of the K562 cell line was downloaded from the ENCODE data portal (https://www.encodeproject.org/) with the accession number ENCSR893RAV.

### U2AF65 eCLIP data and motif analysis

U2AF65 ‘enhanced’ CLIP (eCLIP) data of the K562 cell line was downloaded from the ENCODE data portal (https://www.encodeproject.org/). Before mapping to the transcriptome, the eCLIP reads were processed using the fastx-toolkit. Adapter sequences were trimmed by fastx clipper and low quality reads were filtered using fastq_quality_filter. This analysis required at least 50% of any given read to have a quality score over 30. PCR duplicates were removed by collapsing all the reads (fastx_collapser) and random barcodes were removed. Reads were then mapped using the novoalign software (Novocraft, http://www.novocraft.com) with peak calling executed using Piranha (44).

To obtain the eCLIP read density along different groups of exons, we first obtained the intersection between the eCLIP peaks and 100 nucleotide long regions upstream and downstream of the three groups of exons (αCPs-enhanced, αCPs-repressed and background groups). The coverage was calculated for each nucleotide in the resulting genomic regions (i.e. 100 nucleotide regions 5′ and 3′ of the three groups of exons that have eCLIP peaks). The coverage value represents the number of reads that cover each nucleotide. In order to center the coverage distribution at the presumed eCLIP crosslink site and to avoid the read length shift in the coverage distribution, the eCLIP reads were shifted to center the reads at the presumed crosslink site. The coverage count was normalized to the maximum coverage in each transcript to control for transcript expression levels and avoid having highly expressed transcripts dominate the signal. After this normalization step the coverage for each nucleotide position in a given transcript was represented as a number between 0 and 1. Averaging the corresponding value for each nucleotide position in all the exons and plotting the density curve yielded the distribution of eCLIP reads upstream and downstream of the exon splice acceptor site. The U2AF65 motif scores were calculated by counting all the instances of the U2AF65 motif (TTTTT [C/T]T; as defined by the RNAcompete assay in (12)) in a sliding window of 25 nucleotides in a 100 nucleotide region up- or down-stream of the three groups of exons. When sliding the window by one nucleotide at a time, the motif score was calculated with the following formula:

\begin{equation*} \begin{array}{l} {\rm Motif}\;{\rm score}\;{\rm for}\;{\rm a}\;{\rm given}\;{\rm region} = \\ \frac{\displaystyle{\# \;{\rm of}\;{\rm unique}\;{\rm nucleotides}\;{\rm covered}\;{\rm by}\;{\rm the}\;{\rm motif}}}{\displaystyle{\# \;{\rm of}\;{\rm nuceotide}\;{\rm in}\;{\rm the}\;{\rm window}}} \\ \end{array} \end{equation*}

### Co-Immunoprecipitation (co-IP) and Western blot analysis

K562 cells were washed with ice-cold phosphate buffered saline (PBS) twice and lysed in 1000 μl of ice-cold lysis buffer (50 mM HEPES [pH 7.4], 150 mM NaCl, 1 mM EDTA, 10% glycerol, 1% Triton-X-100, 100 μg/ml RNase A and protease inhibitor) for 30 min at 4°C with rotation. The lysates were centrifuged at 10 000 *g* for 10 min, and the supernatant was collected and used for immunoprecipitation (IP). IP was carried out as described previously (45) with magnetic beads (Novex). IP pellets were dissolved in SDS-loading buffer and were separated on NuPAGE gel and electroblotted to nitrocellulose membranes (Protran BA 85; Schleicher & Schuell) for 1 h at 150 mA in transfer buffer (20 mM Tris, 150 mM glycine, 20% methanol) using a Semi-phor transfer apparatus (Hoefer). The membranes were blocked in 3% nonfat milk in 1x PBS for 1 h at room temperature, followed by an additional hour with primary antisera. Primary rabbit antibody to SF3b145 (A301–605A) was purchased from Bethyl. Anti-U2AF65 antibody, a mouse monoclonal (U4758), was purchased from Sigma. HRP-labeled secondary antibodies (Amersham) were used as detailed by the supplier. Donkey anti-rabbit immunoglobulin G (IgG)-horseradish peroxidase and sheep anti-mouse immunoglobulin G (IgG)-horseradish peroxidase (HRP) secondary antibodies were used at a 1:5000 dilution (Amersham) and signals were visualized by ECL (ECL reagents; Boehringer Mannheim). For quantitative analysis, the primary antibody was visualized with a fluorophore conjugated secondary antibody (IRDye800CW anti-rabbit, #925–32213, LI-COR, Lincoln, NE) and the signal density was measured on a LI263 COR Odyssey CLx Imaging platform.

The IP study quantifying the association with U2A65 with the WT and Mut-2 TARS2 exon 14 (Figure 4D) was carried out on a single-intron minigene constructs (see Minigene analysis, below). Each indicated minigene was individually transfected into K562 cells and corresponding cell extracts were isolated one day post-transfection. IPs with antibodies to U2AF65 (U4758, Sigma) bound to magnetic beads (Novex) were carried out as described previously (45). Pre-immune serum (P.I.) was used as IP control. U2AF65 occupancy at the single splice acceptor in the single exon minigene was quantified by RT-PCR of the co-IP'ed RNA. RT-PCR of GAPDH mRNA was used as background control. The ratio of TARS2/GAPDH was calculated with the WT defined as 1.0.

### EMSA

RNA oligonucleotides were synthesized and 5′-end labeled using T4 polynucleotide kinase (NEB, Beverly, MA, USA) and [γ-^32^P]ATP (Amersham). All labeled oligonucleotides were gel purified on 12% denaturing gels prior to use. Electrophoretic mobility shift assays (EMSA) were carried out as described previously (24) with minor modifications. A total of 5 ng of each probe (∼20 000 cpm) was mixed with 30 μg of K562 S100 extract, The incubation was in 20 μl of binding buffer (10 mM Tris-HCl [pH 7.4], 150 mM KCl, 1.5 mM MgCl_2_ and 0.5 mM dithiothreitol) at room temperature for 20 min. One microliter of heparin (50 mg/ml) was added to each reaction mixture for 10 min prior to loading. Samples were resolved on a non-denaturing 5% polyacrylamide gel.

### RT-PCR

RNAs were treated with DNase I (Invitrogen) and then reverse transcribed using oligo-dT, Moloney murine leukemia virus reverse transcriptase (Promega), and 1x Moloney murine leukemia virus reverse transcription (RT) buffer (Promega) according to manufacturer's instruction. After incubation at 37°C for 1 h, the samples were used as a template for PCR. The forward primer (20 pmol) was end labeled by incubation with [γ^32^ P]ATP. The PCRs included 1 μl of the RT product, 0.2 mM dNTPs, 1.5 mM MgCl_2_, 1 pmol of the labeled primer, 20 pmol of each primer, 0.25 U of AmpliTaq (Perkin Elmer) and 1x PCR buffer II (Perkin Elmer) in a 25-μl reaction. The number of PCR cycles in each study was adjusted to the primers used and originating RNA concentrations. Samples were visualized by 6% denature polyacrylamide gel electrophoresis (PAGE) and quantified by the PhosphorImager (ImageQuant; Molecular Dynamics). Alternative splicing efficiency was determined by calculating the inclusion level (%) of the cassette exon in all isoforms. RT-PCR primers are listed in Table 3 and others from a recent report (13).

### Minigene analysis of alternative 5′ and 3′ splice sites

WT and mutant TARS2 minigenes were cloned into the pI-11(-H3)-PL adenovirus based splicing minigene plasmid. Co-transfections of minigene plasmids and plasmids encoding defined recombinant proteins were performed as described (17). The minigene inserts were PCR amplified with primers (Forward) TCCTTCTGCCTTTGGCCCTA and (Reverse) GAGGCTGGCATATGAAGGGG and cloned onto the vector at the XbaI and XhoI cloning sites. RT-PCR was done with common SP6 and T7 primers. To construct the single-intron minigene, the intron sequence between cassette exon and downstream vector exon was completely removed. The primers (Forward) TCCTTCTGCCTTTGGCCCTA and (Reverse) CTTGAAAGAAAAAAAAAAAAAGTGGATGGAATGGTGGAAGT were used for IP-RT-PCR analysis in Figure 4D.

## RESULTS

### αCPs impact globally on cassette exon splicing

The impact of αCPs on AS was assessed in K562 cells (46). K562 is a Tier 1 ENCODE cell line that has served as an intensively studied model of mammalian gene expression and regulation of hematopoiesis. The workflow of the study is outlined in Figure 1A. Three pools of K562 cells were co-depleted of αCP1 and αCP2 *via* transfection with two distinct siRNAs, each of which co-targeted conserved segments of the αCP1 and αCP2 mRNAs (‘αCP1/2 siRNA’). Three parallel cell pools were transfected with each of three distinct control siRNAs (see Materials and Methods). Effective co-depletion of αCP1/2 proteins was confirmed by Western analysis (representative study; Figure 1B) and corresponding libraries were generated from RNAs isolated from the three αCP1/2 co-depleted pools and from each of the three control pools. The six individually bar-coded RNA-seq libraries were pooled and sequenced in a single flow cell (Illumina Hiseq2000) to achieve a minimum depth of 63 million reads per library (Supplementary Table S1). The RNA-seq data were processed using rMATS (v3.0.7; http://rnaseq-mats.sourceforge.net/, (39,40)) to detect differential AS events triggered by αCP1/2 depletion.

Co-depletion of αCP1 and αCP2 had a significant impact on 1551 AS events in the K562 transcriptome. The inclusion level of alternatively spliced exons was measured as PSI (percent spliced in, or ψ). The 1551 significant AS events showed |ΔPSI| ≥ 0.05 (at least 5% splicing change upon αCP1/2 depletion) and rMATS FDR < 5%. The majority of the 1551 AS events comprised alternative inclusion/exclusion of cassette exons. There were 1051 skipped exon (SE) events involving 919 cassette exons (in some cases, the cassette exons were involved in more than one SE event) (Table 1A). The predominant event following αCP1/2 co-depletion was repression of cassette exon inclusion: the inclusion of 749 exons was repressed in αCP1/2-depleted cells compared with 170 exons being enhanced. These data lead us to conclude that αCP1/2 have a wide impact on splicing within the K562 transcriptome with a predominant impact comprising the enhancement of cassette exon inclusion.

### Cassette exons enhanced by αCP1/2 are selectively flanked by C-rich motifs

The basis for the impact of αCP1/2 co-depletion on cassette exon splicing was assessed by motif enrichment analysis (MEME v4.9.0; see Materials and Methods). The target sequence field for this analysis comprised a 500 bp window centered on the splice acceptor or donor sites bracketing each of the 919 cassette exons impacted by αCP1/2 co-depletion. These data were compared to a background set of 4527 alternatively spliced exons in highly expressed genes (FPKM > 5.0 in at least one sample) that were unaffected by the αCP1/2 depletion (rMATS FDR > 50%). To filter out the those AS events that are almost always skipped or almost always included, the exons in the background set were required to be included at a frequency of >15% in at least one condition (max (PSI-αCP-KD, PSI-CTRL) >15%) (removing exons almost always skipped) and less than 85% in at least one condition (min (PSI-αCP-KD, PSI-CTRL) <85%) (removing exons almost always included).

The positional enrichment of identified motifs in the vicinity of the αCP impacted exons were determined by examining the 500 bp windows in 50 bp bins with step-size of 1 bp and determining the occurrence of each motif within each bin. MEME analysis of the 749 αCPs-enhanced exons revealed a marked enrichment for a C-rich motif ([C/U][C/U][C/U][C/U]C[U/A][C/G]CC[C/U]) (Figure 1C, boxed sequence). This motif was highly enriched immediately adjacent (5′ and/or 3′) to the exons whose splicing was repressed in αCP1/2 co-depleted cells (Figure 1C, red line) and was specifically lacking in the 170 exons whose splicing was increased in αCP1/2 co-depleted cells (Figure 1C, blue line) and in the background exon set (Figure 1C, black line) as well as within the exonic regions (Figure 1C, green rectangle). In clear contrast, the MEME analyses revealed that the two later groups of exons were preceded by canonical U-rich polypyrimidine tract (Figure 1D, boxed sequence) that is present at the majority of mammalian splice acceptor sites and recognized by the PPT binding protein, U2AF65 (Figure 1D, (8)).

MEME analysis was repeated using exon sets that conformed to a higher |ΔPSI| (≥15%). With this more stringent cutoff, there were 362 αCPs-enhanced cassette exons, 56 αCPs-repressed exons and 4742 background exons. The MEME analysis of these exons (Supplementary Figure S1) revealed the same enrichment of a C-rich motif in the αCP1/2 enhanced exon set versus the U-rich motif in the repressed and background exon sets as observed using the |ΔPSI| ≥ 0.05 cutoff. These results lead us to conclude that αCPs selectively enhance inclusion of a subset of cassette exons by binding to an adjacent C-rich sequences overlapping PPT sites. This linkage of αCP function with the C-rich PPT is fully consistent with the binding specificity of αCPs for C-rich polypyrimidine motifs as defined in both *in vitro* and *in vivo* settings (47).

The MEME analysis revealed that, of the 749 αCP1/2-enhanced exons, 92% (686) contained a C-rich intronic motif adjacent to either 5′or 3′ splice site whereas 63 lacked this motif. Of the 686 exons containing C-rich flanking sequences, 94% (644) contained the C-rich motif upstream to the target exon and in 44% (333) this C-rich motif was restricted to this region (i.e. not present 3′ to the exon). In contrast, only 6% (42) had the C-rich motif restricted 3′ to the αCP-enhanced exon (Table 1B). Chi-square analysis of the co-occurrence frequencies of C-rich motifs both 5′ and 3′ to the 749 positively regulated exons, while numerically apparent, did not reach statistical significance (chi-squared = 2.17; *P*-value = 0.141). We conclude from these observations that the great majority of αCP1/2 enhanced exons contain a C-rich sequence that maps 5′ of the adjacent splice acceptor.

Prior studies from our laboratory and others have identified an array of nuclear and cytoplasmic post-transcriptional controls over critical cell functions that are mediated by αCPs. These include cell cycle control (48,49), epithelial to mesenchymal transition (EMT) (27,50–55) and cancer biology (32,50,56–58). With these findings in mind, we performed Ingenuity pathway analysis to determine if αCP1/2 enhanced cassette exons were enriched in specific cellular pathways or biological functions. Ingenuity analysis of the gene set containing αCPs-impacted exons (Table 2A) or the subset of αCP-enhanced exons (Table 2B) revealed enrichment in pathways related to cell death and survival, cellular growth and proliferation, cell cycle controls and RNA post-transcriptional modifications (Table 2). These data support a link between the impact of αCPs on cassette exon splicing and defined roles of the αCPs in fundamental cellular functions and disease processes.

**Figure 2.**
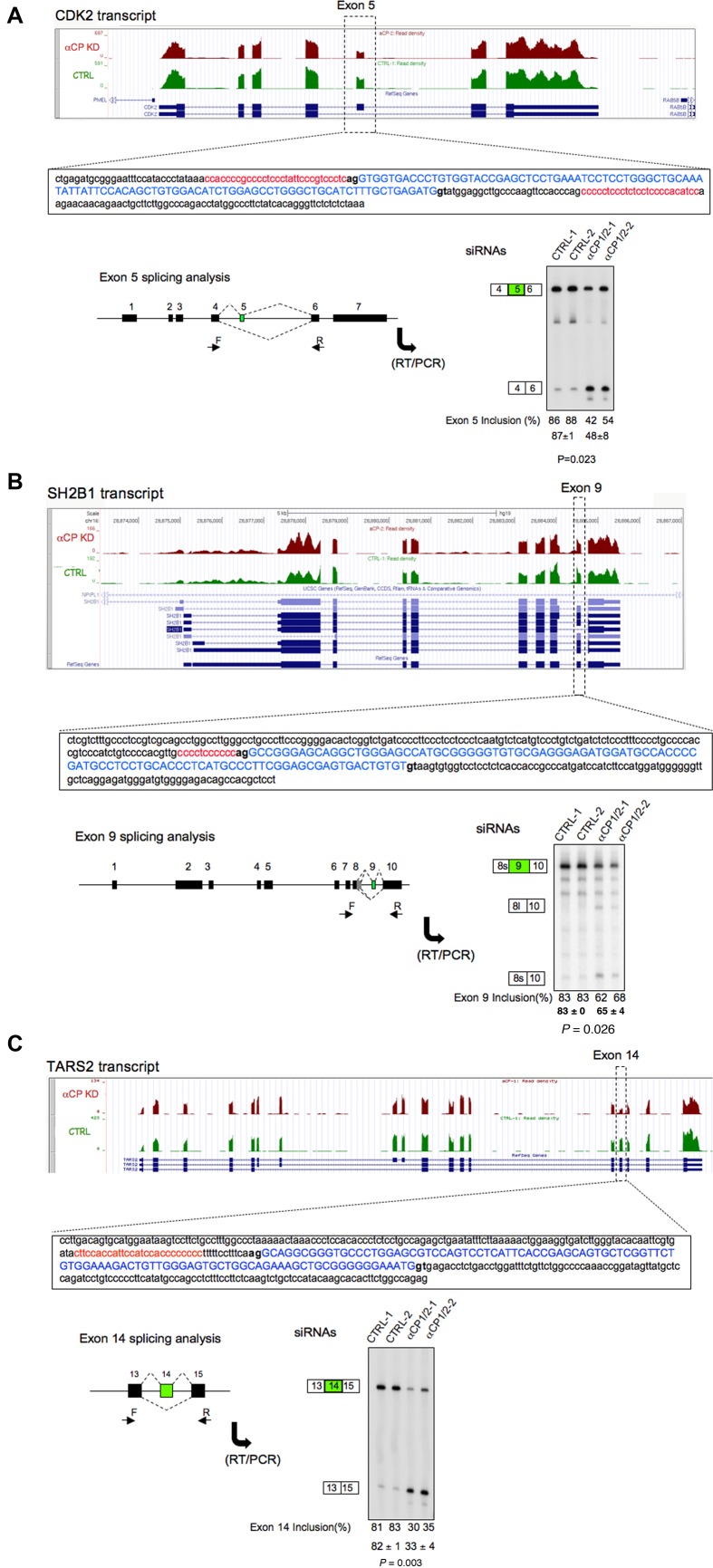
Targeted validations of αCP-dependent cassette exon splicing. (**A**) Inclusion of *CDK2* exon 5 is enhanced by αCP1/2. Top: Genome browser screenshot of *CDK2* RNA-seq data from αCP1/2 depleted cells (brown) and parallel scrambled siRNA-transfected controls (CTRL; green). The cassette exon 5 is enclosed in the dotted box. The blue diagram represents gene structure; exons are shown as boxes and introns as dotted lines. The sequences surrounding the cassette exon impacted by αCP1/2 (exon 5) are shown in an expanded view: exonic sequence (blue uppercase), intronic sequences (lower case) and C-rich regions (red font). The acceptor (ag) and donor (gt) dinucleotides are shown in bold. Bottom left: Diagram of the CDK2 transcript: the impacted cassette exon (green) and the amplimer primers used for targeted analysis (forward and reverse; F and R) are shown below neighboring exons, respectively. The RT/PCR validation study is shown in the adjacent gel; the numbers in the boxes indicate exon number. The cassette exon ‘Inclusion%’ was calculated as the ratio of exon included isoform over the sum of exon-included and exon-excluded isoforms derived from the analysis of two biologic replicates. CTRL-1 and CTRL-2 represent different control siRNAs targeting an unrelated mRNA (GLD-2); αCP(1/2)-1 and -2 are two different siRNAs, each of which co-targets αCP1 and αCP2. *P*-value is shown below the gel. (**B**) Inclusion of *SH2B1* exon 9. Data are organized as in (A), above. (**C**) Inclusion of *TARS2* exon 14. Data are organized as in (A) above.

Three cassette exons containing combinations of intronic C-rich motifs located upstream and/or downstream of the enhanced exon was next validated for αCP dependence by targeted RT-PCR (Figure 2A, B and C). Exon 5 of CDK2 is flanked both upstream and downstream by C-rich motifs, exon 9 of SH2B1 has a C-rich PPT limited to its splice acceptor site, and exon 14 of TARS2 has a C-rich motif that is limited to the splice acceptor site and is situated adjacent to a short U-rich polypyrimidine segment. Eight additional targeted validations of αCP1/2-enhanced exons are shown in Supplementary Figure S2. In all cases, the repression of cassette exon inclusion in αCP1/2-depleted cells was confirmed by the targeted analyses. These data lead us to conclude that the positive regulatory impact (enhancement) of αCPs on cassette exon splicing is mediated *via* αCP binding to corresponding C-rich motifs.

### The two major αCP isoforms, αCP1 and αCP2, individually contribute to enhanced cassette exon splicing

**Figure 3.**
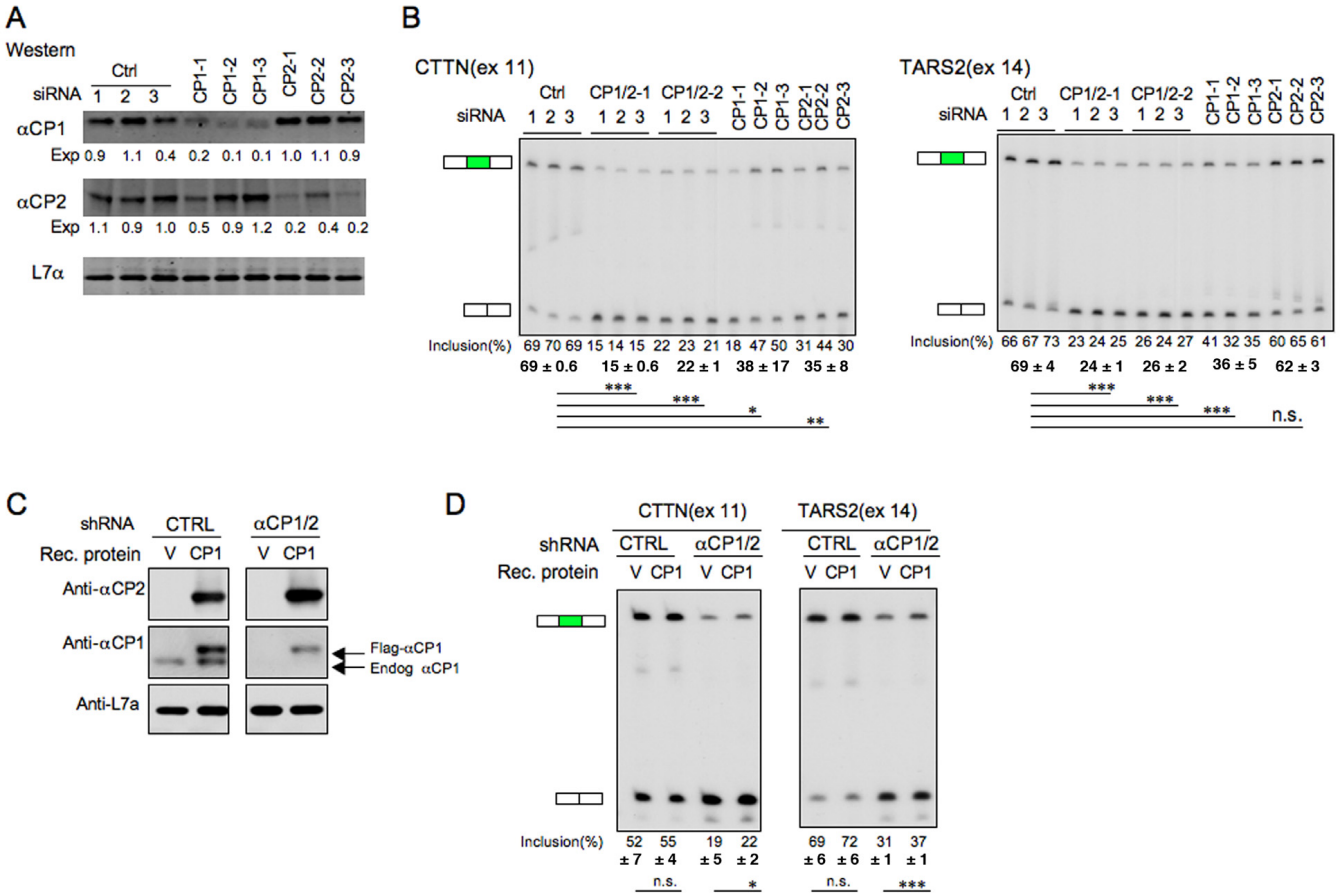
Each of the two major αCP isoforms, αCP1 and αCP2, can individually enhance inclusion of cassette exons flanked by a C-rich splice acceptor site. (**A**) Isotype-specific depletion of αCP1 and αCP2 proteins. K562 cells were individually transfected with three distinct siRNAs that selectively target αCP1 (CP1–1, CP1–2, CP1–3), or αCP2 (CP2–1, CP2–2, CP2–3), or siRNAs co-targeting both αCP isoforms (CP1/2–1, CP1/2–2, triplicate transfections for each siRNA) as well as one siRNA control (triplicate transfections). The cells were assayed three days post transfection by Western blot with isotype-specific antiseras as indicated to the left of each respective panel. Detection of the ribosomal protein, L7a, served as a loading control. The amount of the remaining protein was calculated by comparing with control siRNA knockdown and adjusted to loading control L7a using Odyssey CLx Imaging platform. (**B**) Impact of isotype-specific αCP depletions on cassette exon splicing. Splicing analysis of two cassette exons flanked by C-rich motifs; TARS2 and CTTN. The organization of the figure is as described in Figure 2. The exon inclusion levels (%) in the presence of individual siRNAs to αCP1, αCP2 or Control, are shown below each gel along with corresponding averages and significance values. *P < 0.05, **P < 0.01, ***P < 0.001. (**C**) Western analysis of depletion-repletion assay. K562 cells were co-transfected with control or αCP1/2 shRNAs as indicated, along with vectors expressing recombinant Flag-tagged αCP1 encoded by shRNA-immune mRNAs. V: empty vector; CP1: αCP1 expression vector. Western assay of the cells 3 days post-transfection confirmed the shRNA-mediated depletion and recombinant Flag-αCP1 expression. (**D**) Repletion of αCP1 partially restores exon splicing. Splicing levels (Inclusion%) were assessed for CTTN and TARS2 by RT/PCR as described (Figure 3B). The averages and significance values were shown below each gel (n = 4). *P < 0.05, ***P < 0.001. n.s., not significant.

The preceding data demonstrate that co-depletion of αCP1 and αCP2 enhances the splicing of a subset of cassette exons preceded by C-rich motifs. While αCP1 and αCP2 are known to share a strong binding preference for C-rich motifs and to have extensive functional overlap, they have also been shown to exert a subset of non-redundant functions (28,59–61). We therefore sought to determine whether enhancement of C-rich motif activity was shared by the individual αCP1 and αCP2 isoforms. K562 cells were selectively depleted of either αCP1 or αCP2 using in each case three distinct sets of siRNAs (siRNAs CP1–1, 2 and 3, and siRNAs CP2–1, 2 and 3). Parallel transfections were carried out with siRNAs that co-deplete αCP1 + αCP2 (αCP1/2–1 and αCP1/2–2) and with non-targeting control siRNAs (see Materials and Methods). The isoform-specific depletions were confirmed by Western analyses (Figure 3A) and their impacts were assessed on the splicing of two exons that are repressed in cells co-depleted for αCP1/2: CTTN (exon 11) and TARS2 (exon14) (Figure 3B). We observed strong and equivalent levels of repression of CTTN (exon 11) in cells individually depleted of either αCP1 or αCP2 (Figure 3B) and the combined depletion of both isoforms had an additive impact. For TARS2 (exon 14), the situation was somewhat different in that αCP1 depletion robustly repressed cassette exon splicing while αCP2 depletion had no appreciable effect. In this case the combined depletion of αCP1 and αCP2 was approximately equivalent to that of αCP1 alone. These data lead us to conclude that the two major αCP isoforms, αCP1 and αCP2, can each enhance the splicing of a subset of exons although in certain settings this impact can be isoform specific. The basis for this isoform specificity for the enhancement of certain cassette exon(s) remains unclear and will be the focus of subsequent studies.

The link between αCP function and enhancement of exon splicing was further tested by αCP depletion/repletion. αCP1/2 were co-depleted in K562 cells co-transfected with a plasmid expressing Flag-tagged αCP1 from shRNA ‘immune’ mRNAs (see Materials and Methods). The depletion of endogenous αCP1 and the expression of the recombinant αCP1 protein were both confirmed by Western analysis of the respective co-transfected cells (Figure 3C). Analyses of the αCP1/2 depleted/repleted cells confirmed repression of splicing of CTTN(exon 11) and of TARS2(exon 14) and demonstrated a partial reversal of this repression in the presence of recombinant αCP1 (Figure 3D). These depletion/repletion studies further support the role of αCPs in enhancing the splicing of cassette exons flanked by C-rich sequences.

### Minigene analyses confirm the essential roles of αCPs and the C-rich poly-pyrimidine tract in enhancement of cassette exon splicing

**Figure 4.**
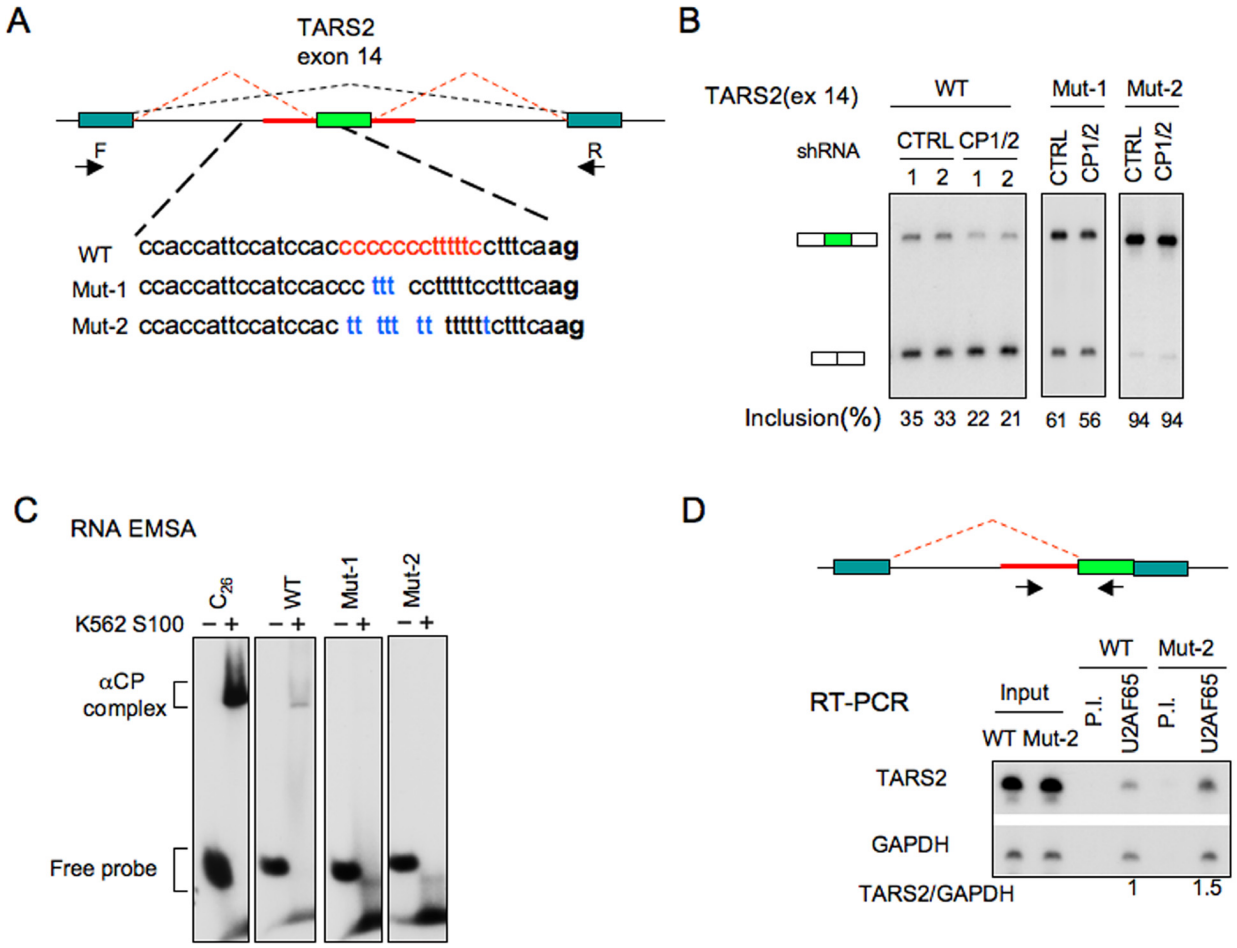
Minigene analysis confirms the impact of C-rich PPT on cassette exons splicing. (**A**) Diagram of TARS2 exon 14 mini-gene. The TARS2 exon 14 (light green rectangle) along with its contiguous intron sequence (red line) was inserted into a minigene test vector containing 5′ and 3′ exons (dark green rectangles) with functional splice donor and splice acceptor sites, respectively. The forward (F) and reverse (R) primers used for the splicing analysis are shown. The C-rich domains in the TARS2 exon14 segment are shown in red and the corresponding sequence substitutions are shown in blue. Intronic sequences in lower case and the acceptor dinucleotide, ag, is bolded. Mutations were introduced into the upstream intron (Mut-1 and Mut-2) to disrupt the integrity of C-rich sequence. These C → U substitutions, Mut-1 and Mut-2 are shown below the WT sequences. Each set of mutations was inserted into the minigene and assessed for its impact on splicing efficiency. (**B**) Mini-gene splicing assay. The WT minigene and each of the two mutant minigenes were co-transfected into K562 cells along with αCP1/2 shRNA or control shRNA. The splicing efficiency of exon 14 is assessed by RT-PCR after a 3-day incubation. (**C**) Impacts of C → U substitutions on αCP complex formation at the PPT are verified by RNA–EMSA. Electrophoretic mobility shift assays (EMSA) for WT, Mut-1, Mut-2 and C_26_ positive control are shown. The positions of the αCP-RNA complex and that of the free RNA probe are indicated to the left of the gel image. (**D**) Occupancy of U2AF65 at the PPT is enhanced by C → U substitutions. The WT and Mut-2 TARS2 exon 14 segments were assembled in corresponding single-intron minigene constructs (top diagram, labeling as in (A)). The WT and Mutant minigenes were individually transfected into K562 cells and cell extracts isolated one day post-transfection were immunoprecipitated with antibodies to U2AF65. Pre-immune serum (PI) was used as IP control. The occupancy of U2AF65 at the splice acceptor was assessed by RT-PCR of the co-IP'ed RNA. RT-PCR of GAPDH mRNA was used as background control. The ratio of TARS2/GAPDH was calculated with the WT set as 1.0.

The relationship of the C-rich PPTs and αCP activity to the control of cassette exon splicing was specifically tested in a series of minigene splicing assays. The alternatively spliced exon 14 of the TARS2 transcript along with its flanking intronic sequences was assembled within an alternative splicing minigene vector (Figure 4A). This TARS2 (exon 14) minigene was transfected into K562 cells along with a plasmid expressing either an shRNA co-targeting αCP1/2 expression or a scrambled shRNA. RT-PCR analysis three days post-transfection revealed that αCP1/2 co-depletion repressed TARS2 (exon 14) splicing (Figure 4B). This response parallels the impact of the αCP1/2 co-depletion on the native TARS2 transcript (Figure 3B), although the degree of impact was less marked in the minigene context.

We next assessed the contribution of the C-rich motifs 5′ to the TARS2 exon 14 splice acceptor to splicing activity by introducing a stepwise reduction in the C/U ratio within the PPT 5′ to exon 14 (Figure 4A, Muts 1 and 2). RNA EMSAs confirmed that C → U substitutions within the PPT resulted in the expected loss of αCP binding (Figure 4C). Minigene splicing analysis revealed that the step-wise C to U conversion (WT → Mut1 → Mut2) resulted in a parallel step-wise increase in exon inclusion (34% → 61% → 94%, respectively). Importantly, these C→ U substitutions resulted in a progressive loss of splicing inhibition subsequent to αCP1/2 co-depletion (Figure 4B). These data support the conclusion that the positive impact of αCP1/2 on exon splicing is dependent on the presence of the C-rich PPT. These data further suggest that the canonical U-rich PPT has a stronger intrinsic splicing activity than the C-rich PPT. This increase in the strength of the *TARS2* (exon 14) splice acceptor subsequent to C → U replacement most likely reflects an enhanced occupancy of U2AF65 at the converted PPT. This was tested by a U2AF65 RNP co-IP in cells expressing a single intron minigene carrying the 5′ terminus and adjacent PPT of the TARS2(exon 14). The study revealed an enrichment for the Mut-2 *TARS2* pre-mRNA in the U2AF65 IP'ed pellet when compared with the WT counterpart (Figure 4D). These data support the model that AS regulation of cassette exons that are preceded by C-rich PPTs bound by αCPs is distinct from splicing control mediated by U2AF65 binding to the canonical U-rich PPT.

### Interactions of αCPs and U2AF65 in control of cassette exon splicing

**Figure 5.**
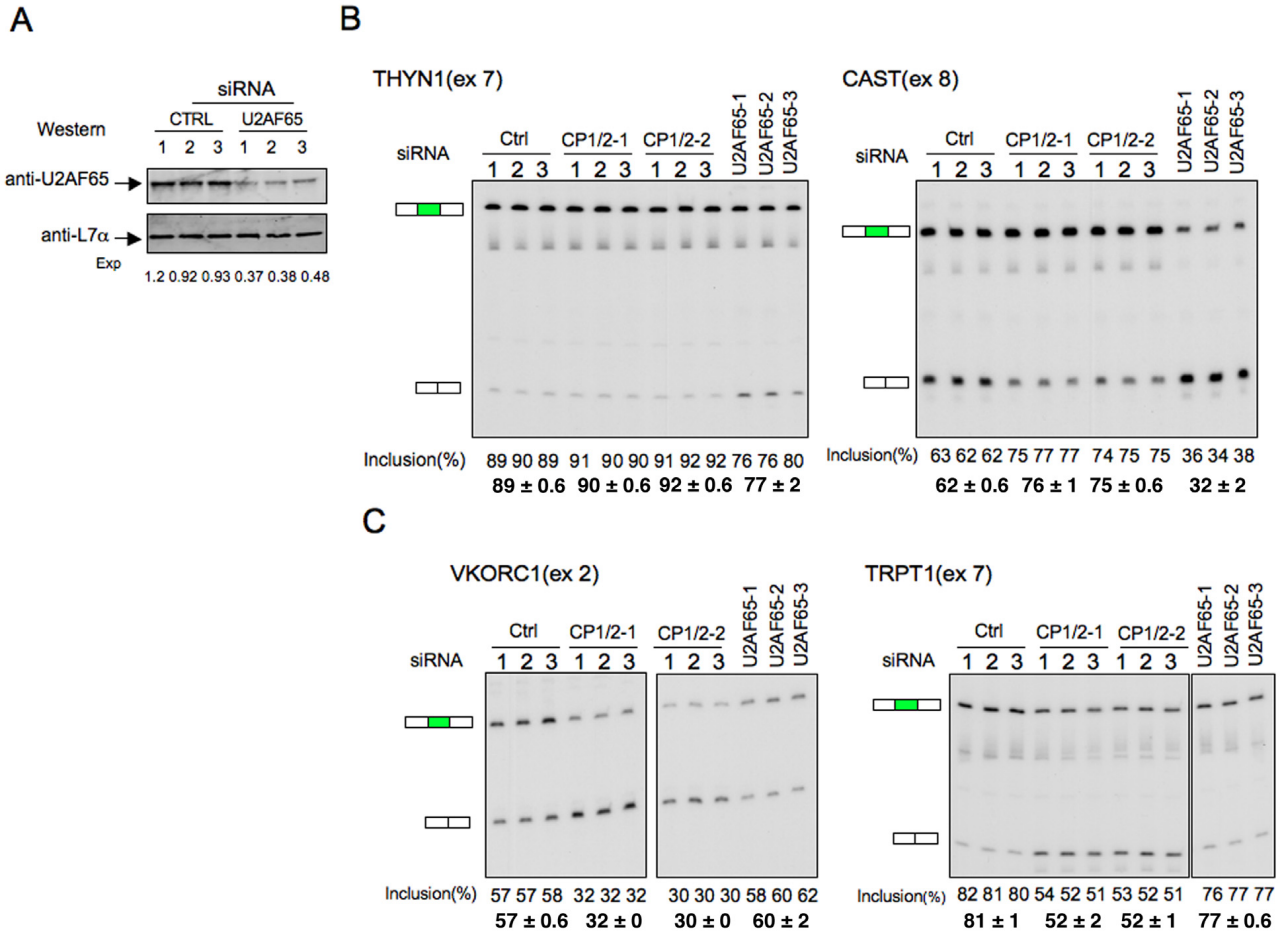
Differential contributions of αCPs and U2AF65 to AS regulation. (**A**) U2AF65 depletion in K562 cells. The indicated control siRNA (triplicates) or 3 independent U2AF65 siRNAs were expressed in K562 cells and assayed 3 days post transfection by Western analysis. The amount of the remaining protein was calculated by comparing with control siRNA knockdown and adjusted to loading control L7a using Odyssey CLx Imaging platform. (**B**) The impacts of U2AF65 and αCP1/2 co-depletion on the splicing of two U2AF65-dependent exons; THYN1(exon 7) and CAST(exon 8). Levels of exon inclusion (%) and averages as determined by targeted RT/PCR are shown below the respective lanes. The analysis reveals negative impact on cassette exon splicing that is specific to the U2AF65 depletion. (**C**) Impacts of selective U2AF65 and αCP1/2 depletions on the splicing of two αCP-sensitive cassette exons; VKORC1(exon 2) and TRPT1(exon 7). RT-PCR analysis data and averages are shown below the respective lanes. The analysis reveals negative impact on cassette exon splicing that is specific to the αCP1/2 co-depletion.

To further distinguish the functions of the αCPs from U2AF65, we assessed the impact of U2AF65 depletion on splicing of αCP-enhanced cassette exons. K562 cells were depleted of U2AF65 using three distinct siRNAs with depletion documented by Western analysis (Figure 5A). As an initial set of positive controls, we assessed the impact of the U2AF65 depletion on four cassette exons which are validated as U2AF65-dependent (13); THYN1 (exon 7), CAST(exon 8), EIF4A2(exon 11) and GSK3B(exon 9). The result demonstrated that the splicing of these four exons was either significantly inhibited (THYN1 and CAST) or enhanced (EIF4A2 and GSK3B) in K562 cells depleted of U2AF65 (Figure 5B and Supplementary Figure S3), consistent with the original report (13). However, these four exons were either unaffected or slightly changed by αCP1/2 co-depletion (Figure 5B and Supplementary Figure S3). A reciprocal response profile was observed in the splicing activities of a set of αCP impacted cassette exons identified in the current study; splicing was repressed in αCP1/2 co-depleted cells while there was no significant impact subsequent to U2AF65 depletion (Figure 5C and Supplementary Figure S4). These data support the model in which C-rich and U-rich sequences at PPTs are respectively regulated by two distinct RNA binding proteins, αCPs and U2AF65.

### αCP isoforms associate *in vivo* with critical splicing factors

**Figure 6.**
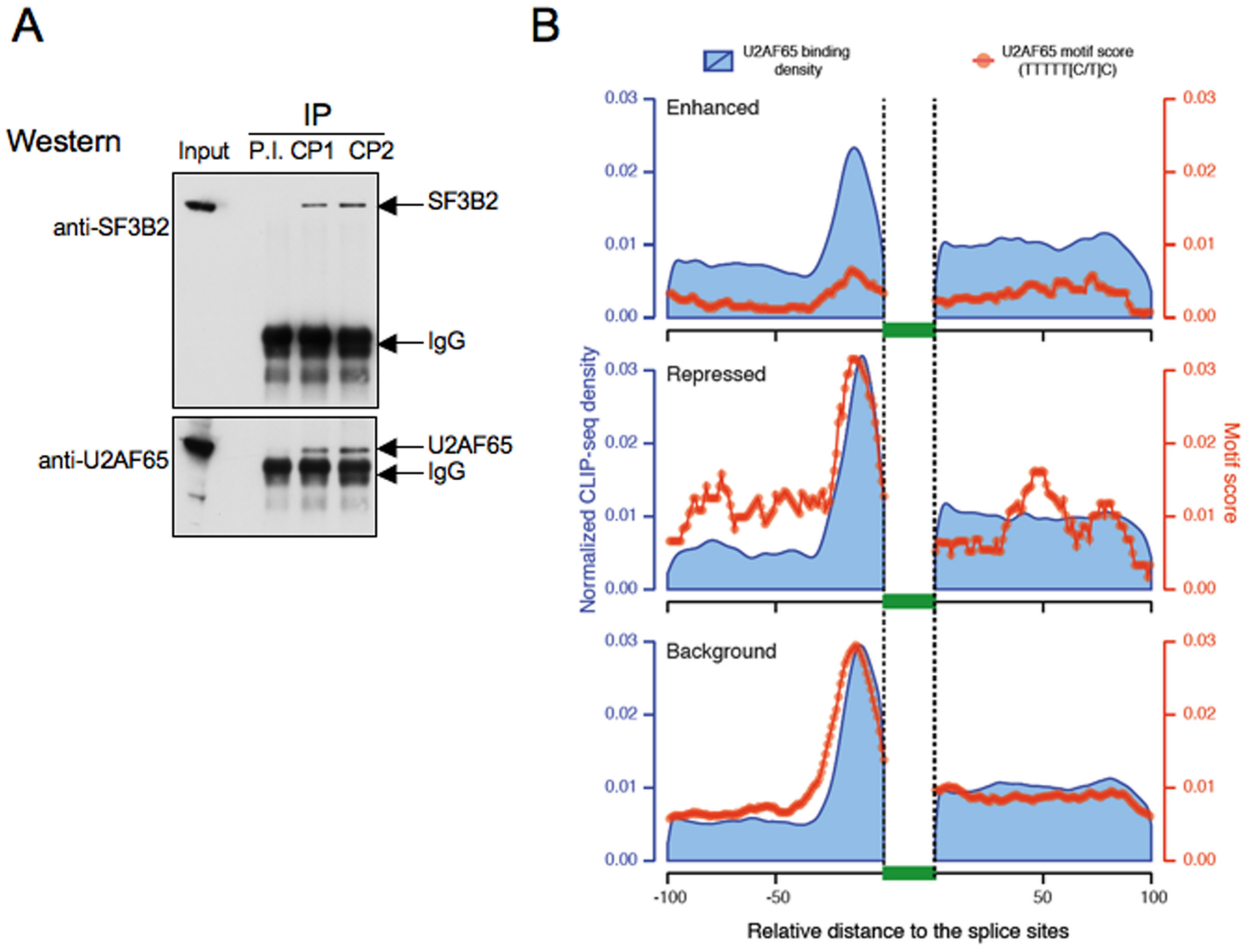
U2AF65 associates with PPTs of αCP-enhanced exons in the absence of its cognate binding site. (**A**) Enrichments of SF3B2–145 and U2AF65 in αCP1 and in αCP2 co-immunoprecipitates. K562 cell extracts were prepared in the presence of RNase A and subjected to immunoprecipitation with isotype-specific antisera to αCP1 and αCP2 in parallel with IP with preimmune serum (PI). Each immunoprecipitate was resolved by SDS-PAGE and then probed (Western blot) with antibodies specific to two splicing factors; SF3B2 and U2AF65. (**B**) Discordance between the distributions of cognate U2AF65 binding sites and U2AF65 occupancy in the regions 5′ to αCP-enhanced exons. K562 U2AF65 eCLIP reads (‘U2AF65 binding density’; blue curves) are displayed upstream and downstream of the three groups of cassette exons; αCPs-enhanced, αCPs-repressed and a defined background set (as defined in Figure 1C and D). The data reveal pronounced occupancy of U2AF65 upstream of all three groups (blue curves). The distribution of the corresponding U2AF65 binding site motifs (TTTTT[C/T]C) (red curves) parallel the binding density 5′ to the repressed and background exons but are dramatically under-represented 5′ to the αCP-enhanced exons. These data suggest that U2AF65 may be recruited to C-rich PPTs upstream of αCP-enhanced cassette exons by an indirect mechanism.

The ability of αCPs to enhance splicing through C-rich motifs at PPT site predicts that they recruit splicing factor(s) that are involved in activation of the splicing pathway. Protein interaction networks suggest that αCPs are involved in spliceosome assembly *via* interaction with the subunit 2 of the splicing factor 3b protein complex, SF3B2–145 (3). Sequence independent binding of the SF3A/SF3B complex 5′ to the branch site is critical to anchoring U2 snRNP to the pre-mRNA and is required for the early stage splicing ‘A’ complex and the ‘E’ complex assembly (8). The association of αCPs with specific splicing factors was tested by co-IP of αCP isoforms from RNase-treated K562 cellular extract. Western analysis revealed that αCP1 and αCP2 IP'ed pellets were both enriched for SF3B2–145 (Figure 6A, top panel). Of additional interest was the observation that both IP'ed pellets were also enriched for U2AF65 (Figure 6A, bottom panel). These data suggest that αCPs’ enhancement of C-rich PPTs is linked to the association of αCPs with both U2 snRNP and U2AF65.

### U2AF65 can be recruited to the C-rich PPTs of αCP-enhanced exons *via* both direct and indirect interactions

The association of αCPs with U2AF65 (Figure 6A) suggested that these proteins may co-occupy subsets of PPTs. To further explore the relationship of αCP with U2AF65, we compared U2AF65 occupancy as mapped in K562 by U2AF65 eCLIP (ENCODE project (62)) and the presence of the U-rich U2AF65 consensus motif (TTTTT[C/T]C) (12) and two closely related U-rich motifs identified by Zagros (63) (Supplementary Figure S5). These comparisons focused on sites flanking αCPs-enhanced cassette exons, sites flanking αCPs-repressed cassette exons, and sites flanking a background exon control set (all groups as defined in our initial transcriptome analyses (Figure 1)). The marked enrichment of the U2AF65 binding motif and U2AF65 occupancy 5′ to the αCP-repressed and background exons (Figure 6B; ‘Repressed’ and ‘Background’) is fully consistent with the canonical model of U-rich PPT recognition by U2AF65 in the great majority of mammalian exons (13). In contrast, the U-rich U2AF65 binding site motif was markedly under-represented 5′ to the αCP-enhanced exons (Figure 6B; ‘Enhanced’). Despite this clear under-representation of the U2AF65 consensus 5′ to the αCP-enhanced exons, the occupancy by U2AF65 at these sites was only minimally diminished when compared to the two other exon groups (Figure 6B; ‘Repressed’ and ‘Background’). The observed discordance between the U2AF65 binding site mapping and the corresponding levels of U2AF65 occupancy suggest that U2AF65 can associate in an indirect fashion with a substantial fraction of C-rich PPTs. Based on the co-IP study (Figure 6A) we propose that this indirect association of U2AF65 at these sites may be mediated by association with the bound αCPs.

**Figure 7.**
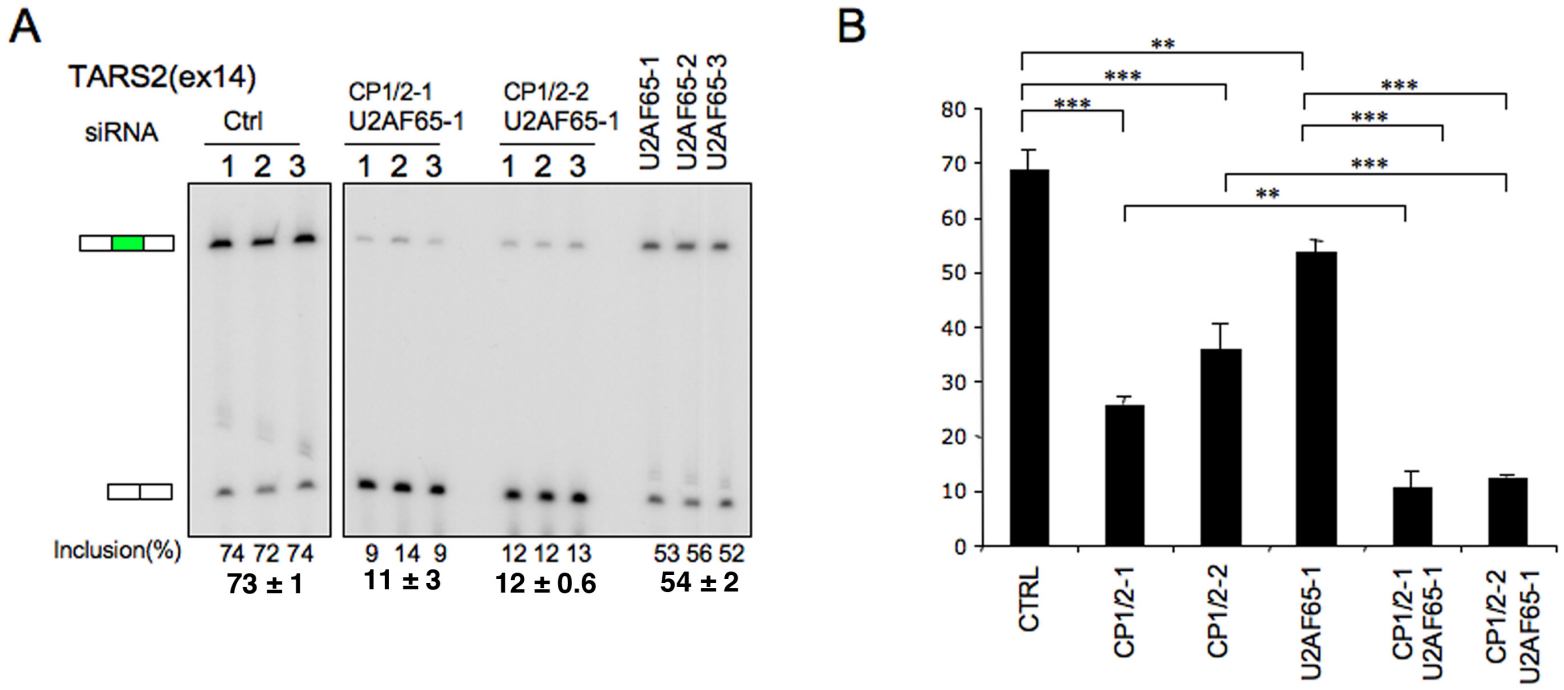
αCPs and U2AF65 can act jointly to enhance inclusion of a cassette exon preceded by a PPT containing adjacent U-rich and C-rich motifs. (**A**) Impact of αCP1/2 and U2AF65 co-depletion. K562 cells were transfected with three siRNA targeting U2AF65 (U2AF65–1, 2 and 3) or were co-transfected with a U2AF65 siRNA along with siRNAs that co-target both αCP isoforms (αCP1/2–1, αCP1/2–2). All studies were carried out in triplicate. The cells were assayed three days post transfection by RT-PCR on the splicing analysis of *TARS2* as described (Figure 3B). (**B**) Quantitative impacts of αCP1/2 depletion, U2AF65 depletion and αCP1/2 + U2AF65 depletion on *TARS2* (exon 14) splicing. αCP1/2 depletion data are from Figure 3B (studies in Figures 3B and 7A were carried out and analyzed entirely in parallel). Averages and SDs are indicated. *P < 0.05, **P < 0.01, ***P < 0.001.

The mapping data (Figure 6B) reveals a minor population of αCP-enhanced PPTs contain U-rich motifs (Figure 6B; small peak in top left panel) and thus may be directly bound by U2AF65. This observation suggests that a subset of exons may be under the combined/cooperative control of αCPs and U2AF65. This model was tested by assessing the impact of combined depletion of U2AF65 and αCP1/2 on the splicing of TARS2(exon 14) (Figure 7A). The PPT preceding this αCP-enhanced exon contains a U-rich segment adjacent (3′) to the defined C-rich motifs (Figure 2C). TARS2(exon 14) splicing is repressed 50% by αCP1/2 co-depletion (Figure 3B) whereas selective depletion of U2AF65 had a smaller but significant repressive effect (73% → 54%)(Figure 7B). Remarkably, the combined depletion of both αCP1/2 and U2AF65 resulted in a dramatic repression of exon inclusion (to 11.5%). The magnitude of this repression substantially exceeds either individual effect (Figure 7B). These data lead us to conclude that, αCPs and U2AF65, while capable of acting independently, can also act in concert to control the alternative splicing of a defined subset of cassette exons.

## DISCUSSION

Controls impacting mammalian mRNA splicing occur primarily at the initial stages of spliceosome assembly on a target transcript (3,8). While several steps can theoretically be targeted for splicing regulation, current evidence points to the binding of U2AF65 to the PPT as an important control point in this process (8), this association of U2AF65 and the PPT can be inhibited, altered or enhanced (11,15). In addition to hnRNPC and PTB (see Introduction), additional RNA-binding proteins can impact on U2AF65 binding and/or function and further highlight the importance of PPT recognition and function on the splicing pathway (3,8). Importantly, two recent genome-wide analyses indicate that U2AF65 only binds to a subset of functional 3′ splice sites. These data suggests that protein factors in addition to U2AF65 can impact on the activity of splice acceptors flanking a substantial fraction of mammalian exons (13,15). Thus divergences in PPT structure and corresponding divergences in locally bound proteins can serve as a basis for specific controls over individual subsets of exons in the mammalian transcriptome.

In this report, we identify 749 cassette exons whose splicing is positively impacted by αCP1/2 RNA binding proteins (Table 1). Analysis of this exon set reveals a marked enrichment for C-rich motifs in their flanking intronic sequences (Figure 1). The most significant site of this cytosine enrichment maps to the PPT adjacent to the 5′ splice site of 644 of the 749 αCP1/2-enhanced exons. The demonstration that αCP1/2 interact with splicing factors associated with the U2 snRNP complex, namely SF3B2 and U2AF65 (Figure 6A), further suggested that the binding of αCPs to C-rich PPTs enhance recruitment of the U2 snRNP and subsequent spliceosome assembly on the target transcript. The binding specificity of αCPs for C-rich sequences is specific and of high affinity (47), consistent with their specific impact on C-rich intronic sequences. This binding activity is clearly distinct from the specificity of U2AF65 for U-rich PPTs. The data in this report demonstrate independent actions of αCPs and U2AF65 in the respective control of C-rich and U-rich subsets of PPTs (Figure 5). Thus we conclude that the involvement of αCPs in splicing regulation diverges from the canonical splicing model that relies on the sole activity of U2AF65.

The *in vivo* association of both αCP1 and αCP2 with U2AF65 (Figure 6A) and the discordance between the mapping of the U2AF65 binding site and U2AF65 occupancy at PPT preceding αCP enhanced exons (Figure 6B), is consistent with a model in which αCPs directly bound at the C-rich PPTs facilitate indirect association/occupancy by U2AF65. The data also reveal that a subset of C-rich PPTs may exist in *cis* with U-rich PPTs, allowing for the coordinated actions of both U2AF65 and αCPs (Figure 7). The actions of these two polypyrimidine-binding proteins is likely to vary widely depending on the structures, sequences and alignments of binding sites within intronic context with corresponding impacts on PPT activity and patterns of alternative splicing.

In summary, we conclude that αCP proteins can enhance the splicing of a defined subset of cassette exons by directly binding to cytosine-rich PPTs and that this αCP-controlled pathway of post-transcriptional control is likely to play a substantial role in the regulation of important cellular function. Genes impacted by αCPs are enriched in basic cellular functions involved in cell death and survival, cellular growth and proliferation and cell cycle control (Table 2), consistent with previously observed positive role of αCPs on cell proliferation (48). Most recently, our studies in mice have revealed that germline inactivation of either of the two major αCP isoforms, αCP1 or αCP2, result in distinctly timed embryonic lethality and that the specific loss of αCP2 has a major impact on differentiation of hematopoietic lineages (64). The mechanisms underlying these phenotypes are likely to reflect the splicing controls highlighted in this report and support essential roles of αCP proteins on alternative splicing regulations critical to an array of development pathways.

## Supplementary Material

SUPPLEMENTARY DATA
