# Supplementary material for: αCP binding to a cytosine-rich subset of polypyrimidine tracts drives a novel pathway of cassette exon splicing in the mammalian transcriptome
Source: Nucleic Acids Res. 2016 Feb 20;44(5):2283–97. doi: 10.1093/nar/gkw088 (PMC4797308; doi:10.1093/nar/gkw088)
Supplement: SUPPLEMENTARY DATA [file supp_44_5_2283__index.html]

αCP binding to a cytosine-rich subset of polypyrimidine tracts drives a novel pathway of cassette exon splicing in the mammalian transcriptome — SUPPLEMENTARY DATA 

# αCP binding to a cytosine-rich subset of polypyrimidine tracts drives a novel pathway of cassette exon splicing in the mammalian transcriptome

## SUPPLEMENTARY DATA

- SUPPLEMENTARY DATA
