# Supplementary material for: αCP binding to a cytosine-rich subset of polypyrimidine tracts drives a novel pathway of cassette exon splicing in the mammalian transcriptome
Source: Nucleic Acids Res. 2016 Feb 20;44(5):2283–97. doi: 10.1093/nar/gkw088 (PMC4797308; doi:10.1093/nar/gkw088)
Supplement: SUPPLEMENTARY DATA [file supp_gkw088_nar-02561-a-2015-File014.pdf]

Supplemental Figures

Supplemental Figure 1. Motif analysis as in Fig. 1C using a |ΔPSI|≥15% (362 αCPs-enhanced cassette exons, 56 αCPs-repressed exons, and 4742 backgorund exons)

Upper panel: Motif analysis of cassette exons whose inclusion was repressed in αCP1/2 co-depleted cells. Cassette exons under-represented in cells co-depleted of αCP1/2 were identified by comparative RNA-seq (**Table 1**). Intronic motifs within 250 bases 'upstream' or 'downstream' of each impacted cassette exon in each of the two categories were assessed by MEME (v4.9.0) for conserved motifs. The displayed level of significance was determined in each case by comparison to a 'Background' set of 4,742 non-impacted alternative exons (rMATS FDR>50%) in highly expressed genes (FPKM>5.0). A C-rich motif (shown in the inset) was identified flanking the αCP1/2-enhanced exons. The graph shows the positional distribution of this motif as assessed by analysis of 50bp bins across the indicated regions in each of the exon groups (αCP1/2-enhanced, αCP1/2-repressed, background).

Lower panel: Motif analysis of exons whose inclusion was not impacted in αCP1/2 co-depleted cells. Analysis as above**.** On the 4,742 background set, the graph shows the positional distribution of the identified U-rich PPT MEME consensus motif as assessed by analysis of 50bp bins across the indicated regions in each of the three exon groups (αCP1/2-enhanced, αCP1/2-repressed, background).

Supplemental Figure 2. Additional targeted validations of cassette exon splicing that is enhanced by αCP1/2

The identity of each locus and the identity of the corresponding amplified region bracketing the impacted cassette exon are indicated to the left of each respective gel. In each case the K562 cells were transfected with each of two independent siRNAs, each of which co-targets αCP1 and αCP2 and with two independent control siRNA. The exon inclusion values were determined as in **Figure 2.** The p-values shown at the bottom were calculated based on the comparison of the two controls and the two sets of αCPs depletions.

Supplemental Figure 3. Impact of depletion of U2AF65 and αCPs on the splicing of a set of U2AF65-dependent exons.

Impact of depletion of U2AF65 (3 independent siRNA) or αCP1/2 (two independent siRNAs) along with a control siRNA (triplicates) on two U2AF65-repressed cassette exons: EIF4A2(exon 11) and GSK3B(exon 9) transcript splicing. Exon inclusion (%) was assessed by RT-PCR.

Supplemental Figure 4. Impact of depletion of U2AF65 and αCPs on the splicing of 8 αCPs-dependent exons.

Impact of depletion of U2AF65 (3 independent siRNA) or αCP1/2 (two independent siRNAs) along with a control siRNA (triplicates) on 8 αCPs-enhanced transcript splicing. Cassette exon inclusion (%) was assessed by RT-PCR.

Supplemental Figure 5. U2AF65 motifs identified by Zagros

Two U2AF65 consensus motifs identified in the K562 U2AF65 eCLIP data set by Zagros.

Supplemental Table 1. RNA-seq mapping statistics

Supplemental Figure 1

Motif search and RNA map with |ΔPSI| ≥ 15%

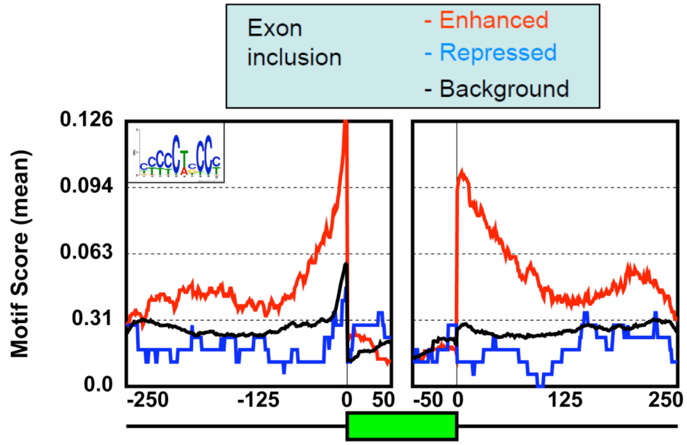

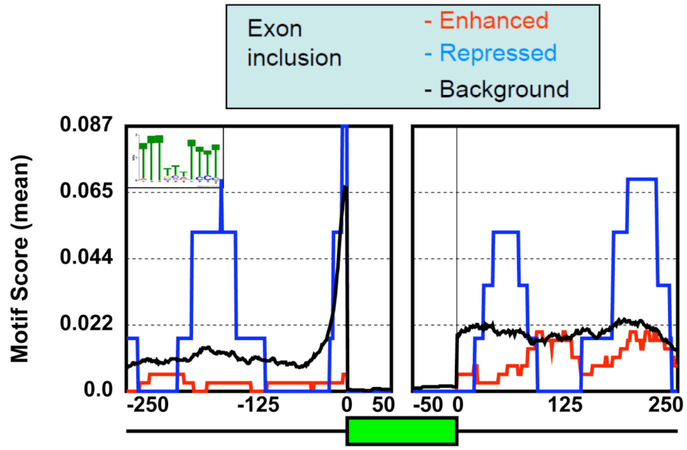

Supplemental Figure 2

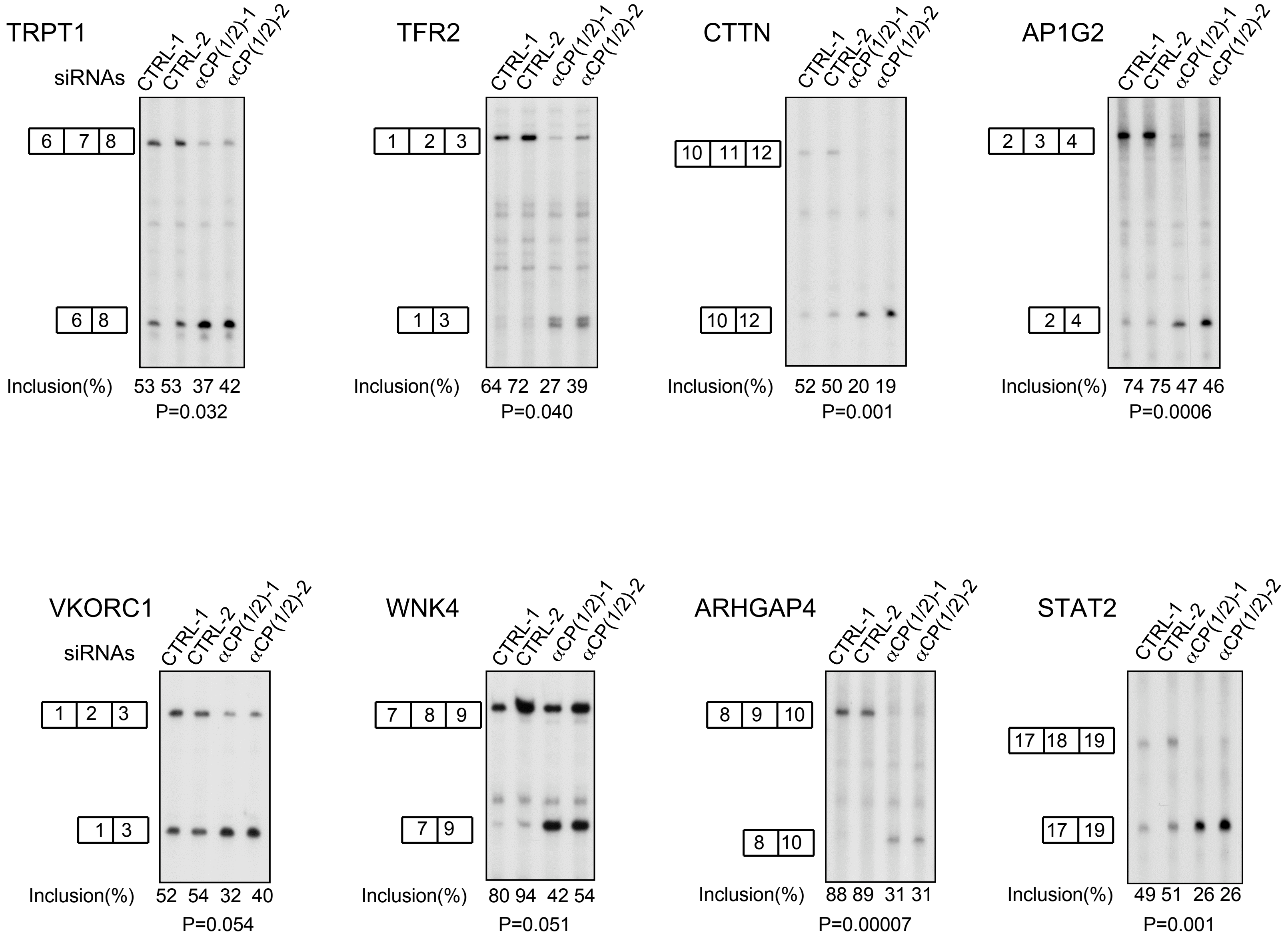

Supplemental Figure 3

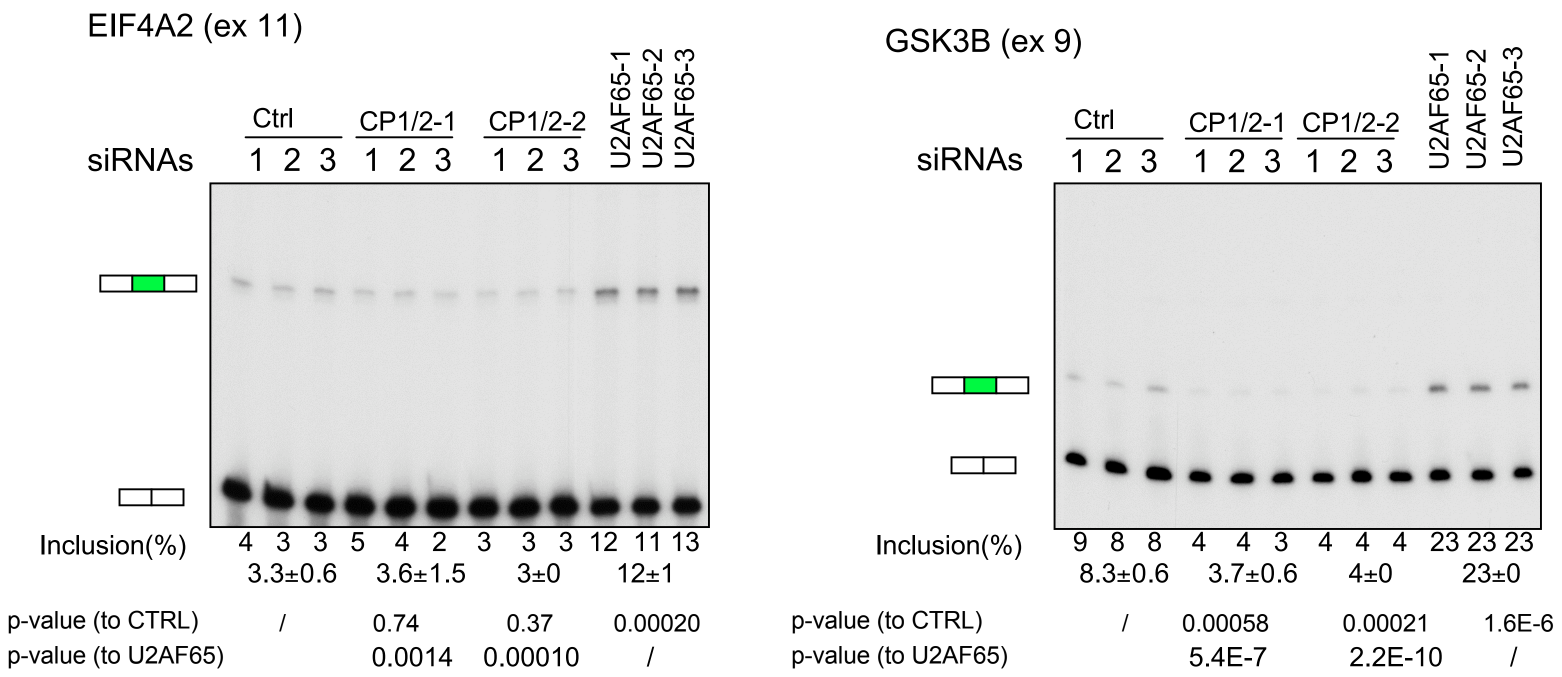

Supplemental Figure 4

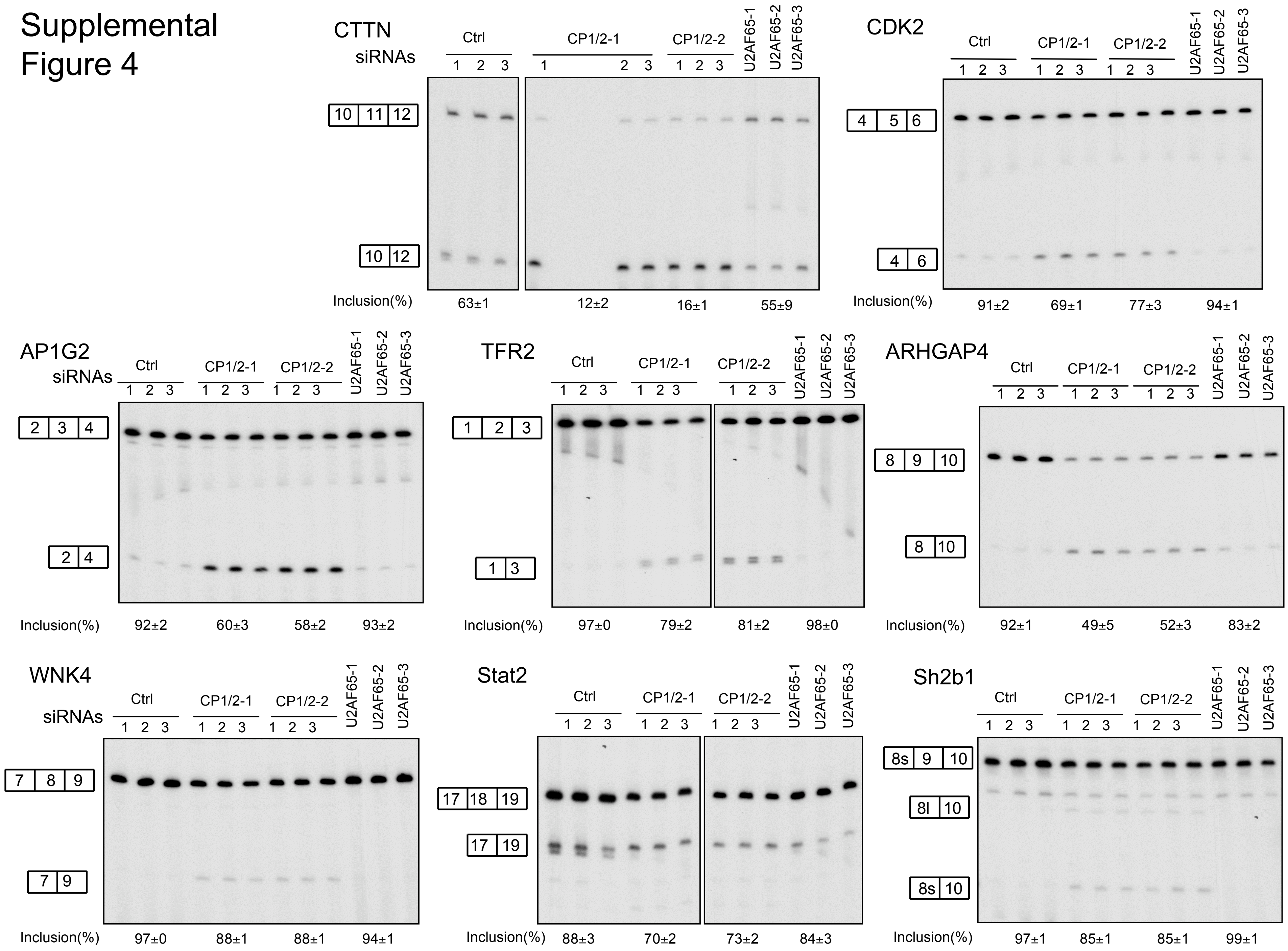

Supplemental Figure 5

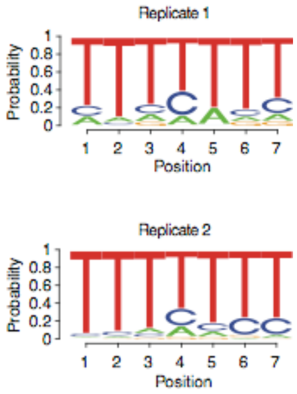

Supplemental Table 1

	total pairs	Mapped Pairs	Mapping Rate	Genome reads	%	Junction reads	%
αCP(1/2)-1	31,641,090	26,235,786	82.9%	32,502,339	51.4%	19,969,233	31.6%
αCP(1/2)-2	45,240,781	37,390,039	82.6%	46,726,928	51.6%	28,053,150	31.0%
αCP(1/2)-3	35,260,235	28,732,391	81.5%	35,542,709	50.4%	21,922,073	31.1%
CTRL-1	39,100,900	32,573,931	83.3%	39,742,432	50.8%	25,405,430	32.5%
CTRL-2	33,844,416	28,114,923	83.1%	34,592,836	51.1%	21,637,010	32.0%
CTRL-3	35,360,110	28,971,820	81.9%	36,665,184	51.8%	21,278,456	30.1%
Total	220,447,532	182,018,890	82.6%	225,772,428	51.2%	138,265,352	31.4%
